# A New Light in Medicine

**Published:** 1917-04

**Authors:** 


### A New Light in Medicine.

A new light in medicine has been discovered by an Englishman named Simpson, and the experiments with it in St. Bartholomew's Hospital in London have been so encouraging that much is expected of it, according to a writer in the *New York Medical Journal,* who says:

"The tendency of modern medicine is to progress away from internal medication. Witness the gradual diminution of the pharmacopeia, the vogue of the psychanalysts, the popularity of nature cures, radium and X-ray, and the marvelous growth of surgery. The influence of sunshine on tuberculosis, the action of the X-rays on malignant growths, and the beneficial effects which hydrotherapy has in certain nervous disorders, are all instances of the replacement of such drugs as creosote, arsenic and the bromides by external therapeutics.

"In line with this movement is the discovery of a new form of light by an English investigator and called after him the Simpson light. This light is obtained from an electric arc formed between electrodes made by a special process from a mixture of the ores of several metals, the chief one being a tungstate of iron and manganese, known as wolfram.

"The chief claim on medical attention of this new light is that it is extremely right in untraviolet wave lengths, meaning obviously different physiological and therapeutic properties. Many experiments have been made at St. Bartholomew's Hospital, London, with the idea of deciding in just which particular field of medicine the Simpson light will find its greatest usefulness.

"It is still too early to generalize, but several interesting discoveries have been made. It seems to stimulate the healing of wounds; shrapnel wounds have been greatly benefited by its treatment. Cases of rheumatoid arthritis and of malignant growth have been treated with little or no appreciable result. Rodent ulcers have been healed by this light. It is believed, however, that its principal indications will be found in certain diseases of the nose and throat.

"Like other similar methods, this treatment is not without its dangers. An erythema (a skin eruption) is often produced and the eyes must be protected or a conjunctivitis (inflammation) will follow. The necessary exposures are very short, which is an added advantage in that a great number of cases can be treated in a short time. So far at least this light seems to be a notable addition to the field of phototherapy."

---

Children from sanitary homes advance more rapidly in school than those from dirty premises.

